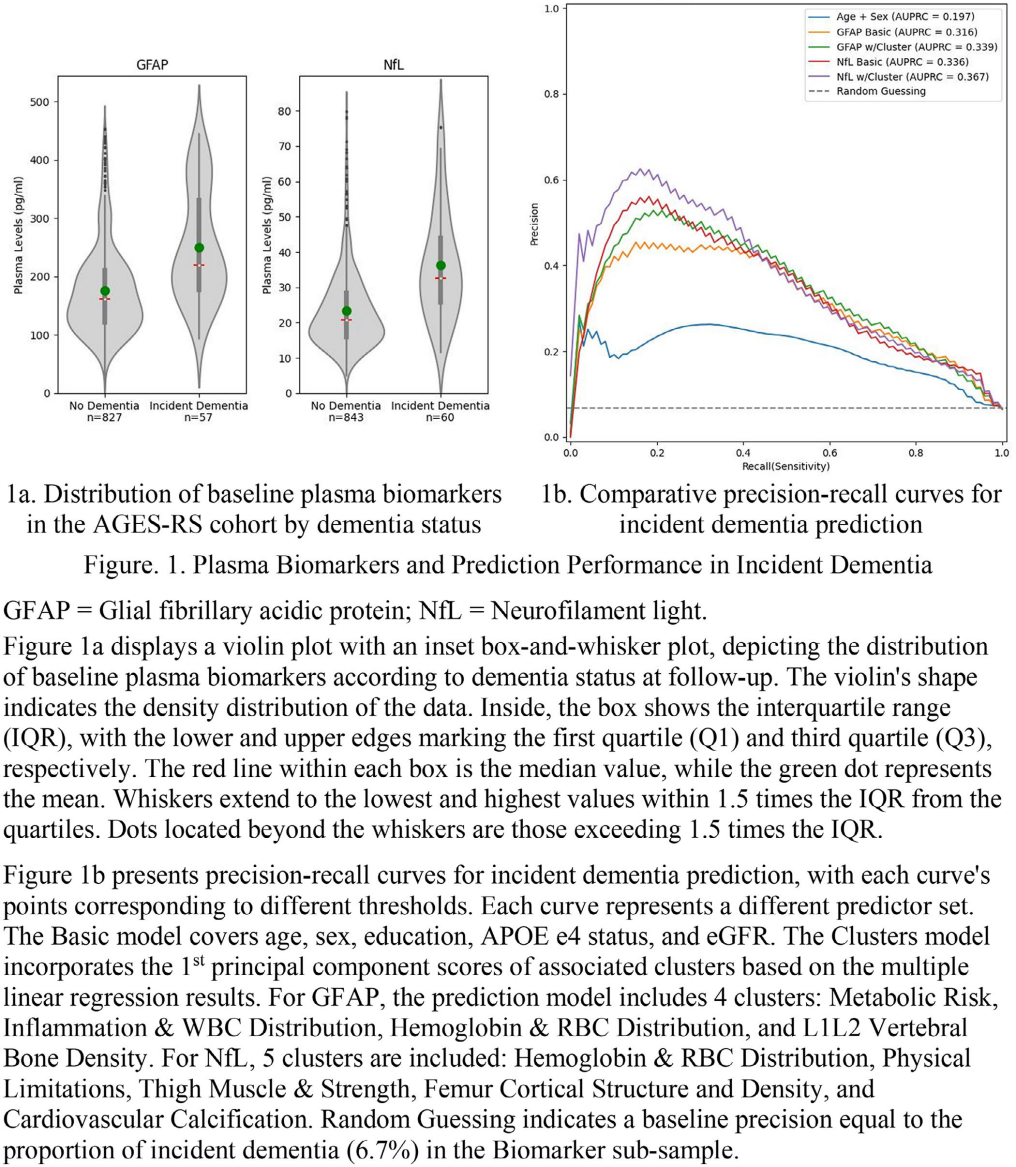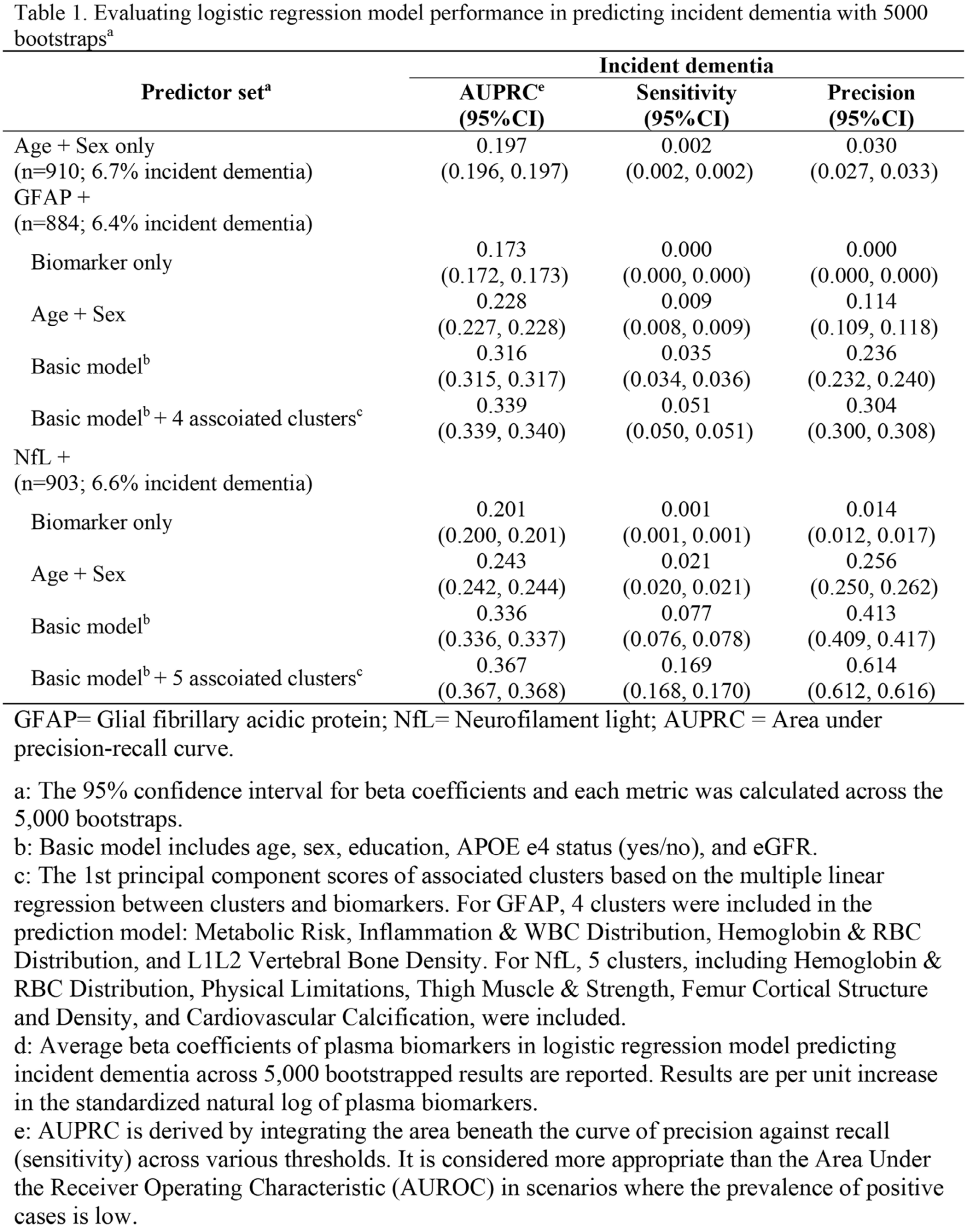# Towards improved dementia prediction in the population: accounting for peripheral factors associated with levels of AD biomarkers

**DOI:** 10.1002/alz.089089

**Published:** 2025-01-09

**Authors:** Yi‐Han Hu, Osorio Meirelles, Claudia L Satizabal, Vilmundur Gudnason, Russell P. Tracy, Sudha Seshadri, Lenore J. Launer

**Affiliations:** ^1^ Laboratory of Epidemiology and Population Sciences, National Institute on Aging, Baltimore, MD USA; ^2^ Glenn Biggs Institute for Alzheimer’s & Neurodegenerative Diseases, University of Texas Health Science Center at San Antonio, San Antonio, TX USA; ^3^ Icelandic Heart Association, Kopavogur Iceland; ^4^ Faculty of Medicine, University of Iceland, Reykjavik Iceland; ^5^ University of Vermont, Colchester, VT USA; ^6^ Glenn Biggs Institute for Alzheimer’s & Neurodegenerative Diseases, University of Texas Health Sciences Center at San Antonio, San Antonio, TX USA; ^7^ Department of Neurology, University of Texas Health Sciences Center, San Antonio, TX USA

## Abstract

**Background:**

Blood‐based biomarkers, glial fibrillary acidic protein (GFAP) and neurofilament light chain (NfL), show potential for dementia risk stratification. Yet, their predictive performance for incident dementia in heterogeneous older population with multi‐morbidities, is not well tested. This study evaluates their predictive ability for incident dementia accounting for differences in factors affecting health.

**Method:**

Data from 910 baseline non‐demented participants of the population‐based Age, Gene/Environment Susceptibility‐Reykjavik Study (AGES‐RS) from 2002 to 2015 (average age 76.6 years, 54.2% female, 6.7% incident dementia) were analyzed. Factors (n = 360) (sociodemographic, clinical, sensory, cardiometabolic, musculoskeletal, medical history, and lifestyle) clustered into 34 groups. Incident dementia cases were identified following the baseline exam until December 2015. Logistic regression models, with 5000 bootstraps, evaluated the predictive power of GFAP and NfL for incident dementia. Predictor sets included: biomarker only; plus age and sex; plus basic factors (education, APOE e4, eGFR); and a full model with associated peripheral clusters. An additional model with age and sex served as the reference for assessing performance without biomarker. Model effectiveness was assessed using area under the precision‐recall curve (AUPRC), precision, and sensitivity (recall), suitable for low dementia prevalence contexts.

**Result:**

Biomarker levels between cases and non‐cases showed significant overlap (Figure 1a). In predictive performance for incident dementia, NfL outperformed GFAP in the biomarker‐only model (AUPRCs, GFAP: 0.17, NfL: 0.20). Integrating peripheral clusters associated with these biomarkers improved the models’ performance, surpassing both biomarker‐only and basic models, especially for NfL (Figure 1b). Precision rates for the final GFAP and NfL models were 0.304 and 0.61, respectively, reflecting approximately 30% and 61% prediction accuracy for incident dementia. The GFAP’s AUPRC improved to 0.339, accurately identifying 5 out of 100 incident dementia cases, while the NfL’s AUPRC was 0.367, predicted 17% of cases.

**Conclusion:**

Our findings indicate that due to the low prevalence of dementia in the general population, using plasma GFAP and NfL as dementia screening tools is not highly effective. However, the addition of peripheral factor clusters does enhance their predictive performance, with NfL having a slightly better performance over GFAP.